# Autonomous assembly and disassembly of gliding molecular robots regulated by a DNA-based molecular controller

**DOI:** 10.1126/sciadv.adn4490

**Published:** 2024-05-31

**Authors:** Ibuki Kawamata, Kohei Nishiyama, Daiki Matsumoto, Shosei Ichiseki, Jakia J. Keya, Kohei Okuyama, Masatoshi Ichikawa, Arif Md. Rashedul Kabir, Yusuke Sato, Daisuke Inoue, Satoshi Murata, Kazuki Sada, Akira Kakugo, Shin-ichiro M. Nomura

**Affiliations:** ^1^ Graduate School of Science, Kyoto University, Kyoto 606-8502, Japan.; ^2^ Graduate School of Chemical Sciences and Engineering, Hokkaido University, Sapporo 060-0810, Japan.; ^3^ Graduate School of Engineering, Tohoku University, Sendai 980-8579, Japan.; ^4^ Faculty of Science, Hokkaido University, Sapporo 060-0810, Japan.; ^5^ Department of Intelligent and Control Systems, Kyushu Institute of Technology, Iizuka 820-8502, Japan.; ^6^ Faculty of Design, Kyushu University, Fukuoka 815-8540, Japan.

## Abstract

In recent years, there has been a growing interest in engineering dynamic and autonomous systems with robotic functionalities using biomolecules. Specifically, the ability of molecular motors to convert chemical energy to mechanical forces and the programmability of DNA are regarded as promising components for these systems. However, current systems rely on the manual addition of external stimuli, limiting the potential for autonomous molecular systems. Here, we show that DNA-based cascade reactions can act as a molecular controller that drives the autonomous assembly and disassembly of DNA-functionalized microtubules propelled by kinesins. The DNA controller is designed to produce two different DNA strands that program the interaction between the microtubules. The gliding microtubules integrated with the controller autonomously assemble to bundle-like structures and disassemble into discrete filaments without external stimuli, which is observable by fluorescence microscopy. We believe this approach to be a starting point toward more autonomous behavior of motor protein–based multicomponent systems with robotic functionalities.

## Teaser

A DNA-based molecular controller regulates the autonomous association/dissociation of gliding microtubules propelled by kinesins.

## INTRODUCTION

Living organisms are autonomous systems capable of sensing their environment, processing information, and executing the necessary actions (*1*–*3*). Inspired by this fascinating autonomy, researchers have been seeking to synthesize autonomous systems that do not require manual operations. Therefore, bioinspired robotics (*4*) has emerged as a field focusing on engineering from hard (*5*) and soft materials (*6*). The extreme miniaturization of bioinspired soft materials has led to molecular robotics (*7*–*9*), aiming to construct robots from molecular components. Biomolecules such as nucleic acids and proteins are promising building block candidates for molecular robots because of their programmability and high specificity (*10*–*12*).

DNA is among the versatile materials for building molecular architectures (*13*–*18*) and designing chemical cascades (*19*–*22*) owing to its ability to arbitrarily tune the reactivity to other DNA by designing the sequences of its building units. Several attempts have been made to develop DNA-based molecular systems with dynamic robotic functionalities, such as assembly lines, autonomous molecular crawling, and reversible cumulative actuation (*23*–*25*). However, slow and small actuation abilities are major drawbacks of DNA-based dynamic molecular robots. Integrating various enzymes, such as DNA polymerase and exonuclease, into DNA strand displacement can accelerate its actuation (*26*, *27*). In contrast, combining relatively larger materials, such as polymers or microparticles, can magnify its motion (*28*–*30*). Yet, overcoming these drawbacks at the same time remains a challenge.

Biomolecular motors have been identified as potential resources to address actuation limitations (*31*). For example, microtubules (MTs) propelled by kinesins (*32*) have been used as powerful molecular motors to drive macroscopic actuators, biosensors, and information processing systems (*33*–*35*). MTs used as molecular actuators are also compatible with DNA systems (*36*), where artificial muscles, spatiotemporal patterning, and active liquid crystals have been proposed (*37*–*39*), although these systems are used only once and are not reversible. To develop reversible molecular robots with fast and large actuation, DNA chemical reactions have been used to programmatically regulate the behavior of DNA-conjugated kinesins or MTs, including vesicle transformation, aster formation, and spatiotemporal pattern control (*38*, *40*–*42*). We have previously investigated the swarming behavior of MTs gliding on a kinesin-adsorbed glass substrate (*43*), reversible examples of which include step-by-step assembly/disassembly and cargo loading/unloading dynamics (*44*–*46*). Despite this important progress, the current generation of reversible MT-kinesin systems are limited in their ability to autonomously perform multiple tasks sequentially, as observed in living organisms, owing to the lack of autonomous control mechanisms suitable for molecular systems.

To maximize the capability of an active molecular system, demonstrating spontaneous temporal behavior without planned external stimuli by combining chemical controller and molecular actuator is of importance. Toward the goal, this study introduces an active molecular system that exhibits autonomous swarming of self-propelled MTs. We used a cascade reaction system as a controller for the gliding MTs, which sequentially generates different DNA strands with a delay. The initially released DNA acts as a cross-linker, causing DNA-functionalized MTs to assemble into bundle-like swarms, and the subsequent DNA removes the cross-links that dissociate the bundles. During the process of the combination, fundamental trial and error was necessary to couple the DNA reactions with MT-kinesin system due to the differences in reaction conditions such as temperature and molecular concentration, which is one of the contributions of this work. After experimental optimization, sequential generation of the DNA strands by the designed reaction cascades was confirmed by gel electrophoresis under practical condition compatible to the MT-kinesin system. Using fluorescence microscopy, we finally demonstrated the autonomous association and dissociation of gliding MTs by the designed controller. This study paves the way for developing autonomous and self-regulated molecular robots.

## RESULTS

### Autonomous association/dissociation molecular system

**Fig. 1.**
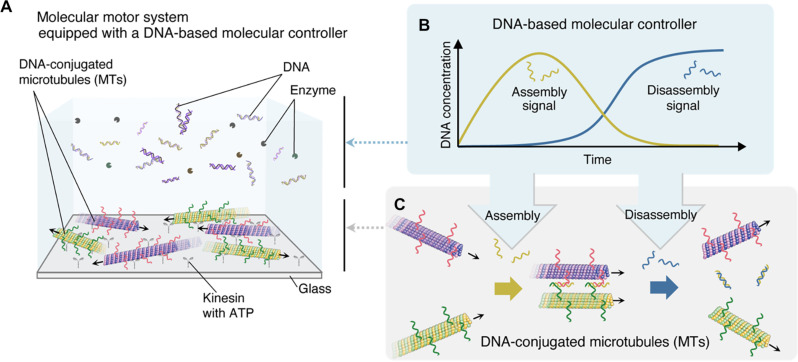
Concept and mechanisms of an autonomous molecular system. (**A**) Experimental setup of an autonomous molecular system. (**B**) Autonomous generations of DNA strands from a DNA-based chemical reaction system that serves as a molecular controller. (**C**) Association and dissociation of MTs propelled on a kinesin-coated glass substrate in the presence of assembly and disassembly signals, respectively.

Figure 1 illustrates the experimental setup of our molecular system, consisting of two key components: kinesin-propelled MTs and a molecular controller. Two types of DNA-conjugated MTs glide on a kinesin-coated glass substrate through adenosine triphosphate (ATP) hydrolysis by kinesin, whereas DNA and enzymes function as a controller regulating MT behavior (Fig. 1A). Our molecular controller, designed on the basis of our previous study (*47*), sequentially generates two DNA strands serving as assembly and disassembly signals (Fig. 1B). Because both MTs are conjugated with single-stranded DNAs with different base sequences, they can associate with each other in the presence of a linker DNA. Dissociation occurs when the duplex is displaced by the newly produced invading DNA (Fig. 1C). Their association leads to the swarming behavior of MTs, while they separate into individual filaments when dissociated.

### Design of a DNA-based molecular controller

First, we developed a DNA-based controller for MTs. The controller was designed to generate two different DNA strands as a result of a series of strand displacement and enzymatic reactions.

**Fig. 2.**
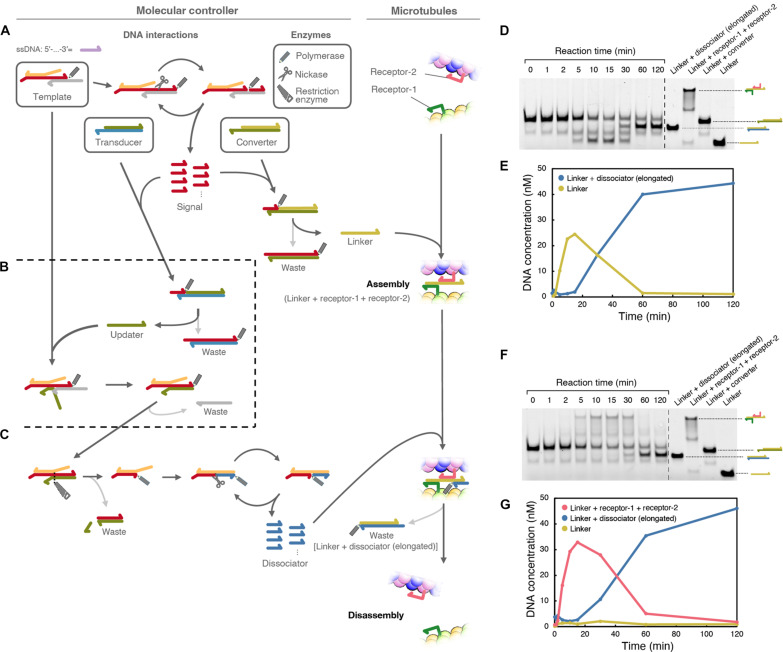
Design of the molecular controller. The system is initially composed of three DNA complexes (Template, Transducer, and Converter) and three types of enzymes (polymerase, nickase, and restriction enzyme), which are surrounded by thick rectangles. Other DNA complexes are intermediate structures. DNA are represented as colored half-head arrows, and polymerase, nickase, and restriction enzymes are represented as pencil, scissors, and saw icons, respectively. Chemical reactions among the DNA complex are connected by black or gray arrows. (**A**) Synthesis of signal and release of the linker. (**B**) Inhibition of the signal synthesis and update of the internal state. (**C**) Dissociator synthesis. (**D** and **E**) Gel electrophoresis of the molecular controller (D) and the time-dependent concentration changes of linker and linker + elongated dissociator (E). (**F** and **G**) Gel electrophoresis of the molecular controller in the presence of receptor DNAs (F) and the time-dependent concentration changes of linker, linker + elongated dissociator, and linker + receptor-1 + receptor-2 (G).

As shown in Fig. 2, the component of the system is three DNA complexes and three types of enzymes. The three DNA complexes are called Template, Converter, and Transducer. In contrast, the three types of enzymes are polymerase, nickase, and restriction enzyme. Polymerase, nickase, and restriction enzymes are protein molecules capable of changing the state of DNA molecules. Specifically, polymerase, nickase, and restriction enzyme can synthesize new DNA from Template DNA, cleave half of the double-stranded DNA, and fully cut the double-stranded DNA. By carefully designing the reaction cascades of hybridization between cDNAs, DNA strand displacement reaction, and enzymatic activities, the molecular controller autonomously performs three basic steps.

The first step starts with the synthesis of the signal, which induces the generation of a linker strand for association with MTs (Fig. 2A). In the first step, the polymerase elongates the DNA strand of the Template, followed by cleavage of the DNA by nickase. Thermal dissociation or strand displacement by polymerase releases a short single-stranded DNA as a signal. The cycle of elongation and cleavage is repeated in the presence of an intact Template, resulting in signal amplification. The signal then hybridizes to the Converter that carries the linker DNA. Elongation of the signal by polymerase displaces the linker from the Converter, leaving the fully duplexed DNA as a waste product. The released linker connects the receptor DNAs conjugated to the MTs, resulting in MT assembly.

The signal then hybridizes with the Transducer to initiate the second step of the operation (Fig. 2B). The hybridization of the signal to the Transducer occurs later than that to the Converter because the complementary region to the signal is shorter in the Transducer than in the Converter. Elongation of the signal by the polymerase releases an updater strand, leaving another waste product. The released updater then hybridizes with the Template and undergoes toehold-mediated strand displacement (*48*). Consequently, the amplification cycle of the first step is stopped.

In the last step of the operations, the linker is removed from the MTs (Fig. 2C). The Template-updater complex forms a recognition site for the restriction enzyme and is cleaved by the enzyme. As a result, a new cycle of elongation and cleavage by polymerase and nickase occurs, producing a new short DNA called dissociator. The dissociator then hybridizes with the single-stranded region of the linker and is subjected to polymerase elongation. Here, the reaction cascade in the second and third steps provides a reasonable delay to keep the MTs assembled for some time before the last reaction. Eventually, MTs disassemble and become individual filaments, producing a waste DNA product containing the linker DNA.

### Sequential strand generation by the molecular controller

The base sequences of DNA strands in the molecular controller were designed using a previously described method (table S1) (*47*). For the receptors, we used 15- and 16-nucleotide-long DNA regions to guarantee stable duplex formation with the linker (fig. S1). The receptors also have poly-T–connecting nucleotides between the hybridizing region and MTs. The estimated melting temperatures of DNA duplexes between “linker and receptor-1” and “linker and receptor-2” were 75.9° and 76.9°C, respectively, using the NUPACK server (*49*). Note that we set the working temperature to 37°C so that the MT-kinesin system and DNA systems could work simultaneously. To further provide compatibility between the DNA and the MT-kinesin systems, we adopted a PIPES-based buffer, which is commonly used for motility assays of MTs, instead of a tris-based buffer, which is commonly used for DNA enzymatic reactions. Through trial and error (table S2 and fig. S2), we found that the activities of the DNA enzymes were maintained by increasing the salt concentration from the typical buffer composition of the motility assay.

To experimentally evaluate the autonomous generation and inactivation of the linker DNA, native polyacrylamide gel electrophoresis was performed without MTs. The time development of the linker DNA concentrations was estimated from the band intensities of the samples incubated for various durations (Fig. 2, D and E). The concentration of single-stranded linker DNA(the lowest bands in Fig. 2D) increased with time after mixing all components of the molecular controller and reached a maximum at approximately 15 min. The concentration gradually decreased and became negligible after approximately 60 min. Alternatively, the concentration of the waste product with the linker [linker + dissociator (elongated)] increased after 15 min and reached a plateau after 60 min, which clearly indicates the autonomous production and inactivation of linker DNA without any external control. Similarly, the connection between receptors was evaluated in the presence of receptor DNAs before conjugation with MTs (Fig. 2, F and G). Although almost the same development time was observed, a slight delay was observed for forming connected receptor DNAs. We also confirmed the immediate generation of the signal and the delayed production of the dissociator using beacon DNAs, which have complementary sequences to the signal and the dissociator (fig. S3).

### Reversible assembly and disassembly of MTs operated by a molecular controller

MTs were polymerized from azide-modified tubulin monomers using a previously described method (see Materials and Methods). Guanosine-5′-[(α,β)-methyleno]triphosphate (GMPCPP) was used as a polymerizing substrate to yield rigid filaments (*50*). We obtained MTs with a diameter of ~25 nm and a mean length of 6.2 ± 2.2 μm (fig. S4). Subsequently, the MTs were conjugated with receptor-DNA1 or receptor-DNA2 through a copper-free click reaction, according to our established protocol (see Materials and Methods and fig. S5A for detailed information). These two DNA strands were labeled with two different fluorescent dyes, FAM (green) and TAMRA (magenta), which enabled us to monitor the DNA-conjugated MTs using a fluorescence microscope (fig. S5B). The labeling ratios of receptor-DNA1 and receptor-DNA2 to tubulin dimers were determined from their absorbance as 68 and 78%, respectively (see fig. S6 for the absorption spectrum and table S3 for the determined concentrations).

To evaluate whether our molecular controller can drive the autonomous assembly and disassembly of MTs, receptor-DNA1– and receptor-DNA2–conjugated MTs were first placed on a kinesin-coated substrate at equal number ratios. DNA-conjugated MTs are propelled by kinesins adsorbed onto the glass surface through ATP hydrolysis. As already known from the literature (*44*), this modification had no adverse effects on the velocity of the MTs. Because a buffer with a higher salt concentration than that commonly used for kinesin is required to drive DNA system by enzymes, we investigated the effect of the salinity of the ambient solution on the kinesin-coated substrate. We found that the direct attachment of kinesin to the glass substrate showed higher stability against salt than when kinesins were adsorbed via streptavidin-biotin interaction (fig. S7). Nevertheless, the number of immobile MTs increased by an adsorption to the glass surface after a long observation (fig. S7). We also quantified the changes in density of MT on the kinesin-coated surface in the absence of DNA and enzymes over time (fig. S8).

**Fig. 3.**
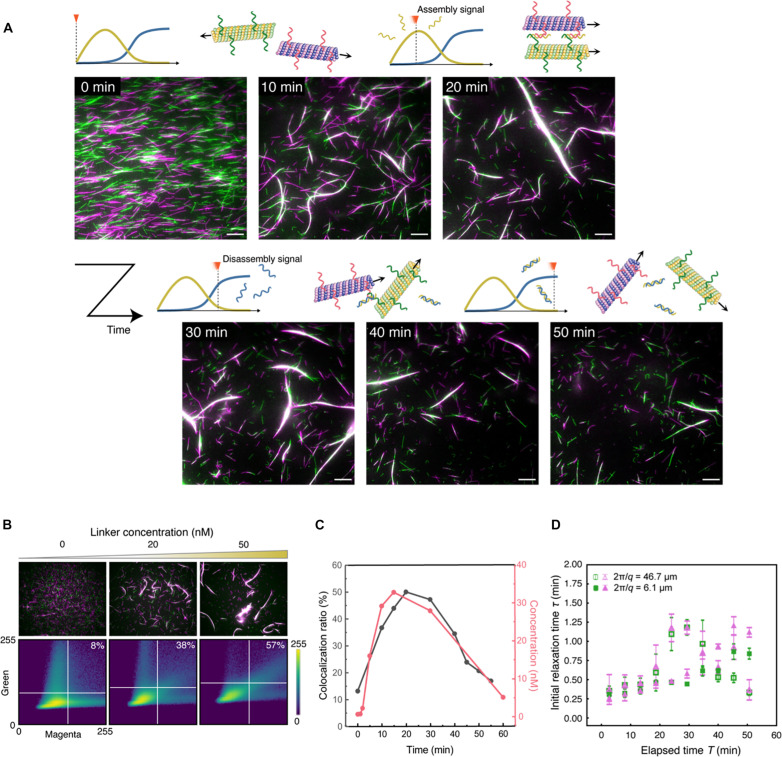
Autonomous association and dissociation of MTs. (**A**) Time-lapse fluorescence microscopy images of the association and dissociation of MTs controlled by a molecular controller (see movie S1). Scale bar, 10 μm. (**B**) Microscopy images of the association of magenta and green MTs under different linker concentrations (top) and the corresponding colocalization ratios (inset) determined from the scatter plots of the intensities of magenta and green pixels (bottom). The microscopy images were captured after 30 min of adding the linker DNA. The colocalization ratio was determined by calculating the proportion of pixels where magenta and green pixels overlapped relative to the total number of pixels meeting specific intensity thresholds. Note that the DNA sequences of the linker and receptor DNAs are different from what we used for the actual molecular controller for the sake of showing the versatility of the linker mechanism. (**C**) Time-dependent colocalization ratios of MTs (black) and the concentration of linker–receptor-1–receptor-2 complex (pink) in the presence of the molecular controller. (**D**) Initial relaxation time τ plotted against elapsed time *T* with wavelengths 2π/*q* = 46.7 μm (hollow) and 6.1 μm (solid), calculated from DMD analysis. The colors of points represent the two channels (green and magenta) of the movie used for the analysis.

Figure 3A shows the fluorescence microscopy images of the MT motility assay in the presence of the molecular controller (movie S1). Initially, the receptor-DNA1 (green)– and receptor-DNA2 (magenta)–conjugated MTs randomly moved without interacting with each other. Within 10 min, the green and magenta MTs started to assemble, forming a swarm in which the green and magenta MTs came close to each other (white in the merged images). The size of the swarms gradually increased as the MTs glided and collided. After 30 min, the swarms started dissociating into green and magenta MTs, and after 50 min, most MTs dissociated and returned to a discrete state. The mean velocities of receptor-DNA1– and receptor-DNA2–conjugated MTs from 0 to 3 min were both 490 ± 20 μm s^−1^ (fig. S9, A and B). The velocities of discrete MTs were almost constant throughout the observation, indicating that the molecular controller did not interfere with the gliding activities of the MTs (fig. S9C). We were also able to change the timing of disassembly by tuning the concentration of restriction enzyme, which demonstrate the programmability of our system (fig. S10).

To quantify the assembly/disassembly performance of MTs, we computed the colocalization ratio of the green and magenta pixels from the image at each time point (Fig. 3B). The colocalization ratio was defined as the ratio of the number of white pixels to the number of green, magenta, and white pixels. We made a scatter plot to illustrate the frequencies of magenta and green pixel intensities in a microscopy image (see Materials and Methods for details). The validity of the colocalization ratio was tested in independent experiments by varying the linker concentrations (Fig. 3B).

The colocalization ratios determined over time are plotted as black dots in Fig. 3C. The ratio started from about 13% and increased up to 20 min of observation, reaching a maximum of 50%. After 20 min, the ratio started decreasing, reaching 21% at 50 min. This result quantitatively confirms the transient assembly of green and magenta MTs in the presence of the molecular controller. This temporal change was in good agreement with the concentration changes of the linker–receptor-1–receptor-2 complex determined by gel electrophoresis (plotted in pink in Fig. 3C). The correspondence between them strongly suggests that the observed assembly and disassembly were due to the production of linker and dissociator DNA by the enzymatic reactions.

To characterize the dynamics of swarming MTs, we analyzed the movie using the differential dynamic microscopy (DDM) method (*51*, *52*) for both green and magenta images (Fig. 3A). From DDM data, we calculated the relaxation times for the length scales that correspond to discrete filament and swarm of MTs. The DDM analysis gave relationships between lag time and intermediate scattering function for each time interval starting from different frames (fig. S11). In each graph, the first four frames (including *F* = 1, which is the value at lag time = 0) of the obtained intermediate scattering function for each wave number were fitted with an exponential function. The relaxation time τ was then defined as the time that takes for the intermediate scattering function to decay to 1/*e* and was calculated by extrapolation or interpolation from the fitting parameters. The relaxation times obtained for the elapsed time were averaged over a certain time duration (over every 16 frames) and plotted for each corresponding interval (Fig. 3D and fig. S12). The above procedure was performed for each of green and magenta images of the movie, the graphs of which showed a similar tendency.

As shown in Fig. 3D, the relaxation time corresponding to the single filament length almost monotonically increased with respect to time. The increase in the relaxation time indicates that motility of the discrete filament gradually decreased. In contrast, the actual velocity of the single filament directly measured from the movie was constant (fig. S9). From the movie, the increment can be explained by the nonspecific absorption of some filaments to the substrate. On the other hand, the relaxation time of the data using the length scale of the swarm of MTs showed a similar trend until approximately 20 min, whereas the value started to increase and then dropped after 30 min, finally returning to the initial state. The trend indicates that the dynamic structure that corresponds to the wavelength appeared and disappeared over time, having good agreement with the design of the system.

## DISCUSSION

This study demonstrated the autonomous association and dissociation of gliding MTs using a DNA-based molecular controller (Fig. 1). The experimental results showed that a DNA chemical reaction system can sequentially generate two types of DNA strands with a sufficient delay, which can hybridize and dehybridize the two receptor DNAs (Fig. 2). Last, by introducing the designed molecular controller into the DNA-conjugated MT system, we successfully controlled the assembly and disassembly of MTs gliding on a kinesin-coated surface without an external control (Fig. 3). The integrated system clearly showed that the molecular controller made of DNA drove reversible state transitions in the behavior of the MT biomolecular motor system. The transitions were quantitatively measured by color and DDM analysis, indicating that the green and magenta MTs colocalized and formed large motile structures. The structures were observed from approximately 20 to 40 min and disappeared after that (Fig. 3, C and D).

However, some limitations to the functionality of the proposed system exist. For example, this study did not address changing the delay between autonomous association and dissociation. Moreover, the autonomous association and dissociation of receptor DNAs can occur only once in our system. Installing DNA-based oscillators is one possible solution for repeating association and dissociation cycles (*53*). Although conventional DNA oscillators can control the behavior of stable materials such as polystyrene beads (*29*), slow oscillation is not suitable for fragile materials such as MTs with short lifetimes. The short lifetime of the MT-kinesin system (approximately 2 hours) is caused by the detachment of MTs from the kinesin-coated glass surface (fig. S8). We anticipate that the cause of detachment is the hindrance of the interaction between MTs and kinesins, which might result from high salt concentration and temperature. However, this limitation could be overcome by using thermostable kinesin extracted from thermophilic fungi (*54*) and further optimizing the buffer composition. Nevertheless, our rapid molecular controller has the potential to enable fragile MT systems to respond immediately to changes in the surrounding environment.

As an issue for future research, the DNA circuit designed in this study has an output with a delay and signal amplification; however, the state of MT gliding as an actuator is not explicitly fed back. Molecular programming of the DNA circuits to respond according to the state of the actuated system, such as the thickness of the bundle, magnitude of the load, and difference in signal concentration, would provide smarter behavior of swarming molecular robots. By solving these issues, harnessing an autonomous molecular system for nano-micro technological applications (*55*) will be our next challenge. The proposed system offers a new concept of autonomously controlled materials, which we named “auto-matter” realized and driven by molecules and equipped with a smart controller that encodes the instructions of the system.

## MATERIALS AND METHODS

### Sample preparation and gel electrophoresis

All DNAs, except receptor DNAs, were purchased from Eurofins Genomics (Japan). The receptor DNAs were purchased from Nihon Gene Research Laboratories (Sendai, Japan). The substock of Template, Converter, and Transducer were prepared by annealing the sample from 80° to 25°C at −1°C/min using an Eppendorf (Germany) thermal cycler in 1 μM concentration. All the enzymes, which are Klenow fragments (3′-5′ exo-) for polymerase, Nt. BbvCI for nickase, and EcoRI for restriction enzyme were purchased from New England Biolabs (Ipswich, MA, USA). A premix of enzymes was prepared at a 2× concentration on ice and mixed with the sample only when necessary to guarantee their sufficient activity. For the experiments shown in fig. S3, the beacon concentration was 100 nM. To inactivate the activities of the enzymes, samples for gel electrophoresis were kept at 85°C for 20 min and cooled down to 25°C at a rate of −1°C/min after incubating for predefined time. For the polyacrylamide gel electrophoresis, a hydrogel was prepared by mixing 12% acrylamide (acrylamide monomer to BIS was 29:1; Serva Electrophoresis GmbH, Germany) in 1× tris borate EDTA (TBE) buffer (NIPPON GENE, Japan), 0.1 wt % ammonium persulfate (FUJIFILM, Japan), and 0.1% (vol %) tetramethylethylenediamine (FUJIFILM, Japan). After the samples were applied, the gel in 1 × TBE buffer (Takara, Japan) was subjected to a constant voltage (200 V) for 40 min at room temperature (25°C). After the application of voltage, the gel was directly observed to observe the fluorescent-labeled DNA without dyeing using a ChemiDoc MP gel imager (Bio-Rad, USA). The concentration of DNA was characterized by band intensities compared to a control sample using ImageJ software.

### Purification of tubulin and kinesin and labeling of tubulin

Tubulin was purified from porcine brain through two cycles of polymerization and depolymerization using a high concentration of PIPES buffer (1 M PIPES, 20 mM EGTA, and 10 mM MgCl_2_) and then preserved in BRB80 buffer (80 mM PIPES, 1 mM EGTA, and 2 mM MgCl_2_, pH adjusted to 6.8 using KOH) (*56*). Recombinant kinesin-1, consisting of the first 573 amino acid residues of human kinesin-1, was prepared as previously described (*57*). Azide-labeled tubulin was prepared using N_3_-PEG_4_-NHS following an established protocol for labeling tubulin with a fluorescent dye (*58*). Tubulin concentration was determined by measuring the absorbance at 280 nm using an ultraviolet (UV) spectrophotometer (Nanodrop 2000c).

### Preparation of DNA-conjugated MTs

MTs were prepared by adding azide tubulin to polymerization buffer (80 mM PIPES, 1 mM EGTA, 1 mM MgCl_2_, and 1 mM GMPCPP, pH adjusted to 6.8 using KOH) to a final concentration of 56 μM tubulin incubating at 37°C for 30 min. Copper-free click reaction was initiated by adding 3.5 μl of dibenzocyclooctyne-conjugated receptor-DNAs (500 μM) to the 5 μl of azide-labeled MTs (56 μM), which allowed azide-alkyne cycloaddition reaction and incubated at 37°C for 6 hours (*59*). Cushion buffer (100 μl) (BRB80 buffer supplemented with 60% glycerol) was used to separate the MTs by centrifugation at 201,000*g* (S55A2-0156 rotor, Hitachi) for 1 hour at 37°C. After removing the supernatant, the pellet of receptor-DNA–conjugated MTs was washed once with 100 μl of BRB80P (BRB80 supplemented with 1 mM taxol) and dissolved in 15 μl BRB80P.

### Measurement of the labeling ratio of receptor-DNA to tubulin dimers

The receptor DNA–conjugated MTs were depolymerized into receptor DNA–conjugated tubulins by keeping them on ice overnight. The absorption spectra of the receptor-DNA–conjugated tubulin dimers were measured using a spectrophotometer (NanoDrop 2000c, Thermo Fisher Scientific Inc.) and deconvoluted using the normal distribution function in Microsoft Excel (Microsoft Corporation) with peaks at 260 and 280 nm. The concentrations of receptor-DNAs and tubulin dimers were calculated from the Beer-Lambert law using the molar extinction coefficient of tubulin dimers (115,000 liter mol^−1^ cm^−1^) and receptor-DNAs (receptor-DNA1 = 265,000 liter mol^−1^ cm^−1^ and receptor-DNA2 = 249,000 liter mol^−1^ cm^−1^), from which the labeling ratio was determined.

### Demonstration of swarming of MTs

A flow cell with dimensions of 9 mm by 2.5 mm by 0.45 mm (L by W by H) was assembled from two cover glasses (MATSUNAMI Inc.) using a double-sided tape as a spacer. The flow cell was filled with 5 μl of casein buffer [DNA reaction buffer (80 mM PIPES, 60 mM NaCl, 7 mM MgCl_2_, and 80 mM KOH, pH adjusted to 6.8 using NaOH) supplemented with 0.5 mg mL^−1^ casein]. After incubation for 3 min, 0.3 μM kinesin solution was introduced into the flow cell and incubated for 5 min. After washing the flow cell with 5 μl of wash buffer (DNA buffer supplemented with 0.5 mg ml^−1^ casein, 1 mM dithiothreitol, and 10 μM taxol), 5 μl of MT (TAMRA-labeled receptor-DNA1–conjugated MTs and FAM-labeled receptor-DNA2–conjugated MTs) solution was introduced and incubated for 2 min. After washing with 10 μl of wash buffer, 5 μl of motility buffer (wash buffer supplemented with 5 mM ATP, 4.5 mg ml^−1^ glucose, 50 U ml^−1^ glucose oxidase, 50 U ml^−1^ catalase, 1 mM trolox, 100 nM input mix, 10 nM linker mix, 500 nM Gate mix, 2.8 mM deoxynucleotide triphosphates, 300 U ml^−1^ Klenow fragment (3′-5′ exo-), 800 U ml^−1^ Nt.BbvCI, and 1500 U ml^−1^ EcoRI) was introduced. The time of ATP addition was set at 0 min. Soon after adding the motility buffer, the MTs were monitored using a fluorescence microscope at 37°C.

### Fluorescence microscopy

The samples were illuminated with a 100 W mercury lamp and visualized using an epifluorescence microscope (Eclipse Ti, Nikon) with an oil-coupled Plan Apo 60× numerical aperture 1.4 objective (Nikon). UV cutoff filter blocks [tetramethyl rhodamine isothiocyanate (TRITC): EX 540/25, DM565, BA605/55; green fluorescent protein B (GFP-B): EX460-500, DM505, BA510-560; Nikon] were used in the optical path of the microscope. Images were captured using a cooled complementary metal-oxide semiconductor camera (NEO sCMOS, Andor) connected to a computer. Two neutral density (ND) filters (ND4, 12.5% transmittance for TRITC, and ND1, 25.0% transmittance for GFP-B) were inserted into the illumination light path of the fluorescence microscope to reduce the photobleaching of the samples.

### Data analysis

Fluorescence microscopy images were analyzed using NIS-Elements AR 5.2 (Nikon) and Fiji-ImageJ 1.53 s software (National Institutes of Health, USA). The velocity of the gliding MTs was measured using the ImageJ plugin “MTrackJ” (https://imagej.net/MTrackJ).

### Estimation of colocalization ratio

Colocalization was calculated from snapshots of a movie using a simple algorithm (*60*). We defined the threshold to distinguish the signal from the background as the mean plus the SD of the brightness distribution for each green and magenta channel. If the magenta and green brightness of a pixel exceeds the thresholds in both the magenta and green channels, it is regarded as a white pixel. If only the magenta or green channel exceeds the threshold, it is regarded as a magenta or green pixel, respectively. If none of these exceeds the threshold, it is a background pixel. Let M, G, and W be the number of pixels regarded as magenta, green, and white, respectively. The colocalization ratio is computed as W/(M + G + W).

### DDM analysis

We used DDM analysis to calculate relaxation times for the length scales of interest from the movies (*51*, *52*). We divided the movie, consisting of 180 frames of images, into 155 sets, each containing 25 successive frames of images. The time corresponding to the midpoint of the 25 frames is taken as the elapsed time *T* of the dataset. DDM analysis was then performed on each image set to obtain the intermediate scattering function *F*(*q*, *t*) over the 25 frames. As a typical wavelength for the analysis, we used 6.1 and 46.7 μm for single filament and swarm, respectively. The program for the DDM analysis was the one provided in (*61*–*63*). The first four frames (including *F* = 1, which is the value at lag time = 0) of the obtained intermediate scattering function for each wave number were fitted with an exponential function. The initial relaxation time τ was defined as the time it takes for the intermediate scattering function to decay to 1/*e* and was calculated by extrapolation or interpolation from the fitting parameters. The initial relaxation times obtained for the elapsed time were averaged over every 16 frames and plotted for each corresponding interval. The above procedure was performed for each of green and magenta images.

## Supplementary Materials

### This PDF file includes:

Supplementary Text Figs. S1 to S12 Tables S1 to S3 Legend for movie S1 References
