# Supplementary material for: Autonomous assembly and disassembly of gliding molecular robots regulated by a DNA-based molecular controller
Source: Sci Adv. 2024 May 31;10(22):eadn4490. doi: 10.1126/sciadv.adn4490 (PMC11141615; doi:10.1126/sciadv.adn4490)
Supplement: Supplementary file 1 — Supplementary Text Figs. S1 to S12 Tables S1 to S3 Legend for movie S1 References [file sciadv.adn4490_sm.pdf]

Science Advances
AAAS

Supplementary Materials for

Autonomous assembly and disassembly of gliding molecular robots regulated by a DNA-based molecular controller

Ibuki Kawamata *et al.*

Corresponding author: Akira Kakugo, kakugo.akira.8n@kyoto-u.ac.jp;
Shin-ichiro M. Nomura, shinichiro.nomura.b5@tohoku.ac.jp

Sci. Adv. **10**, eadn4490 (2024)
DOI: 10.1126/sciadv.adn4490

The PDF file includes:

Supplementary Text
Figs. S1 to S12
Tables S1 to S3
Legend for movie S1
References

Other Supplementary Material for this manuscript includes the following:

Movie S1

Supplementary Text

1. Investigation of the effect of salinity on a kinesin-coated substrate

We have explored how the salt concentration in the surrounding medium affects a kinesin-coated substrate. We incubated kinesin-coated substrates in aqueous buffers of various salt concentrations and assessed their conditions by performing a gliding assay of MTs.

The flow cells were prepared with two different methods: direct attachment and via streptavidin-biotin.

In the direct attachment, casein buffer (BRB80 (80 mM PIPES, 1 mM $MgCl_2$, 1 mM EGTA, pH maintained to 6.8 using KOH) supplemented with casein (0.5 mg mL^{-1})) was added to the flow cell and incubated for 3 min. After washing with 5 µL of wash buffer (BRB80 buffer supplemented with 1 mM dithiothreitol, casein (4.5 mg mL^{-1}), d-glucose (4.5 mg mL^{-1}), glucose oxidase (50 U mL^{-1}), catalase (50 U mL^{-1}), 1.0 mM $MgCl_2$, and 10 µM taxol), 1.0 µM kinesin solution was introduced into the flow cell. Then, the flow cell was filled with wash buffer prepared using different mixing ratios of BRB80 and DNA reaction buffer (80 mM PIPES, 80 mM KOH, 60 mM NaCl, 6 mM $MgCl_2$, pH maintained to 6.8 using NaOH) and incubated for 0, 90, and 180 min. After the incubation, the flow cell was washed with 5 µL of wash buffer, and 5 µL of green MT (without DNA modification) solution was introduced and incubated for 2 min, followed by washing with 5 µL of wash buffer. The green MTs were polymerized from a mixture of 56 µM tubulin (80% (v/v)) and ATTO 488 dye-labeled tubulin (20% (v/v)). Preparation of ATTO488-labeled tubulin was performed using ATTO488 NHS ester following the established protocol (*58*). The motility of MTs was initiated by applying 5 µL of ATP buffer (wash buffer supplemented with 5 mM ATP and 0.2% methylcellulose (w/v)).

In the via streptavidin-biotin method, 5.0 µL of biotin BSA buffer (BRB80 supplemented with biotin BSA (1.0 mg mL^{-1})) was added to the flow cell and incubated for 3 min. After washing with 20 µL of BRB80, streptavidin buffer (BRB80 supplemented with streptavidin (1.0 mg mL^{-1})) was introduced into the flow cell and incubated for 5 min. After washing the flow cell with 15 µL of BRB80, 5.0 µL of casein buffer was added and incubated for 5 min. After washing with 10 µL of BRB80, 5.0 µL of 1.0 µM biotinylated kinesin was introduced into the flow cell. We used a biotinylated kinesin consisting of the first 465 amino acid residues of human kinesin-1 with an N-terminal histidine tag, and a C-terminal avi-tag which was prepared as described in previously published reports by partially modifying the expression and purification methods (*64*). Similarly to the direct attachment, the flow cell was supplied with BRB80, DNA buffer, or their mixture and incubated for certain times. After washing with 5.0 µL of wash buffer, 5 µL of green MT (ATTO488-labeled MTs) solution was introduced and incubated for 2 min, followed by washing with 5 µL of wash buffer. The motility of MTs was initiated by applying 5 µL of ATP buffer (wash buffer supplemented with 5 mM ATP and 0.2% methylcellulose (w/v)).

The time of ATP addition was set as 0 min. 5 min after the addition of ATP buffer, MTs were monitored using an epifluorescence microscope at room temperature (25 °C).

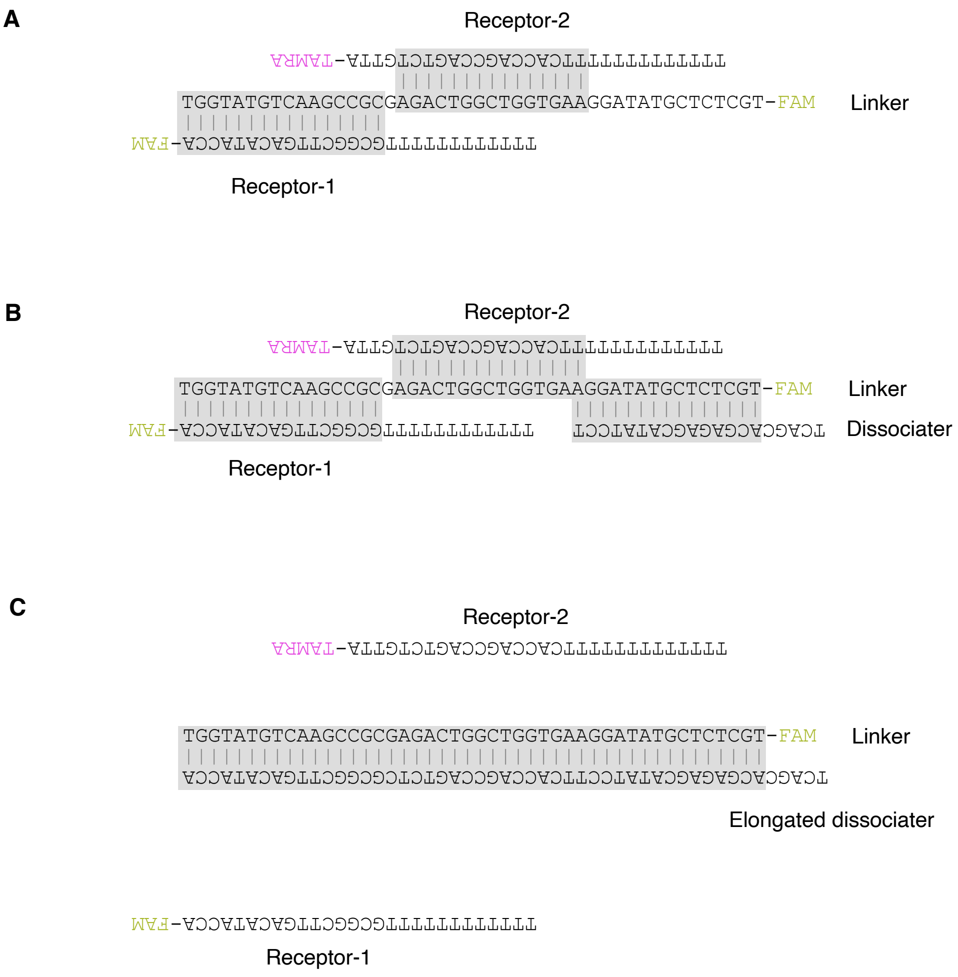

Fig. S1. Hybridization of linker DNA with receptor and dissociator DNAs. (**A**) Hybridization of linker with receptor DNAs. (**B**) Hybridization of dissociator with linker DNA crosslinking the receptor DNAs. (**C**) Duplex formation of linker and its complementary strand (elongated dissociator) and concurrent removal of the Receptor DNAs from the linker.

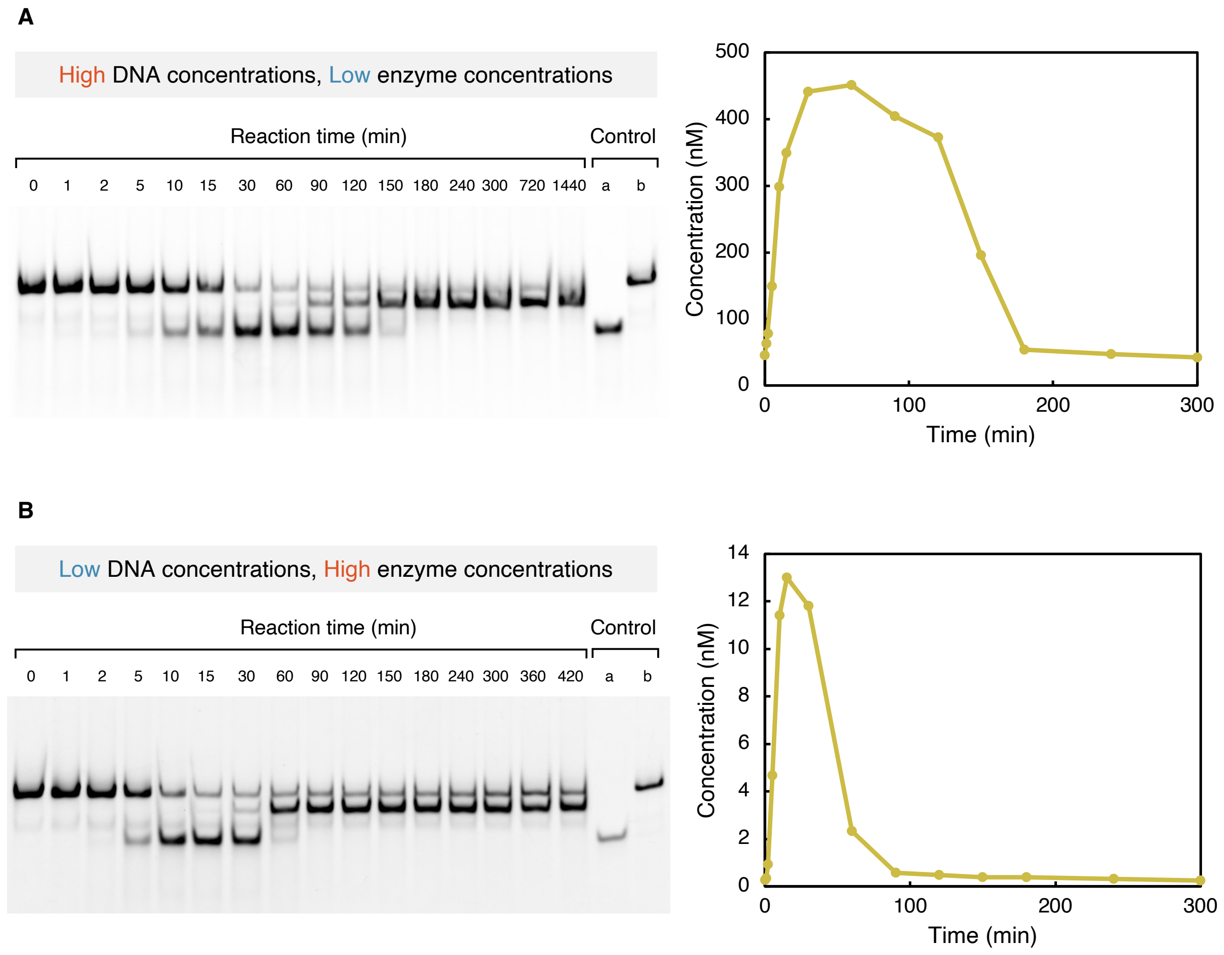

Fig. S2. Effect of buffer composition on the kinetics of the molecular controller. (**A**, **B**) Time-dependent concentration changes of linker in Tris buffer (with 50 mM NaCl and 6 mM $MgCl_2$, neutralized with 10 mM acetate) in (A) high DNA concentrations (200 nM input mix; 500 nM linker mix; 200 nM update mix) and low enzyme concentrations (300 U mL^{-1} polymerase; 800 U mL^{-1} nickase; 1500 U mL^{-1} restriction enzyme) and (B) low DNA concentrations (100 nM input mix; 20 nM linker mix; 100 nM update mix) and high enzyme concentrations (450 U mL^{-1} polymerase; 1200 U mL^{-1} nickase; 2250 U mL^{-1} restriction enzyme) estimated from gel electrophoresis. Controls in gel images: linker (lane a), linker + transducer (lane b). The time required to terminate whole reactions became shorter by decreasing the concentration of DNA and increasing the concentration of enzymes. We further optimized the buffer composition by changing to PIPES-based buffer (detailed composition shown in Method) to improve the compatibility to gliding assay.

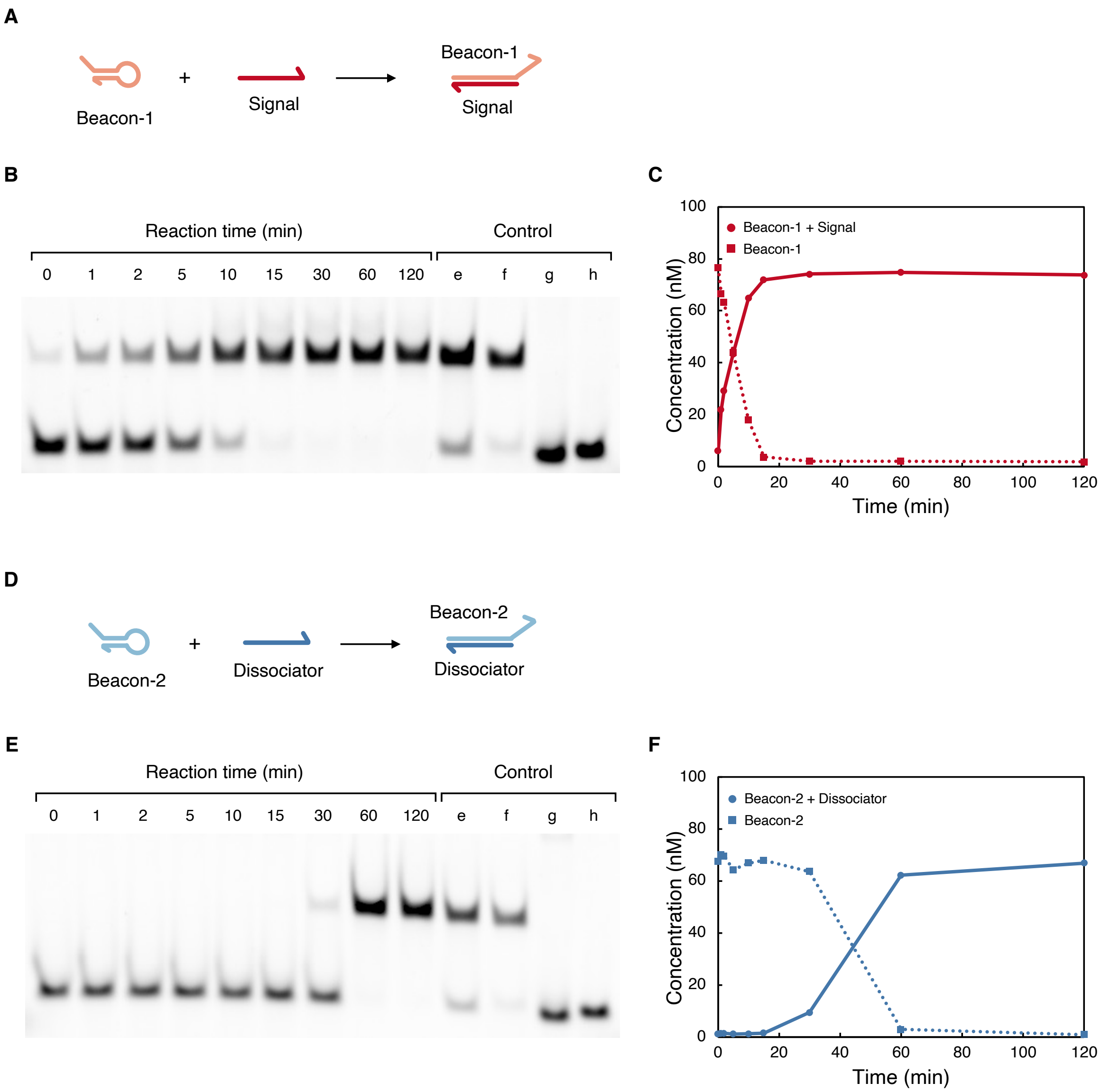

Fig. S3. Generation of signal and dissociator. (**A**) Hybridization of beacon-1 and signal. (**B**) Gel electrophoresis in the presence of beacon-1 to confirm the generation of signal. Control: beacon-2 + dissociator (lane e), beacon-1 + signal (lane f), beacon-2 (lane g), beacon-1 (lane h). (**C**) Time-dependent concentration changes of beacon-1 and beacon-1 + signal. (**D**) Hybridization of beacon-2 and dissociator (**E**) Gel electrophoresis in the presence of beacon-2 to confirm the generation of dissociator. Control is the same as (B). (**F**) Time-dependent concentration changes of beacon-2 and beacon-2 + dissociator.

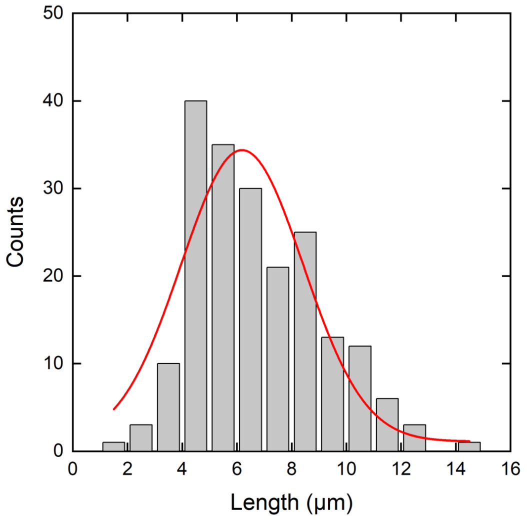

Fig. S4. Distribution of the length of MTs. The number of MTs considered for analysis was 100.

The distribution of MT length was fitted to the Gaussian equation ($y = y_0 + \frac{A}{w\sqrt{\pi/2}} e^{-2\frac{(x-x_c)^2}{w^2}}$) for normal distribution. The fitted curves are represented by the solid lines (R^2 = 0.81). The mean length obtained from the fitting was 6.2 ± 2.2 μm. The arithmetic mean of the length of MTs was 6.8 ± 2.3 μm.

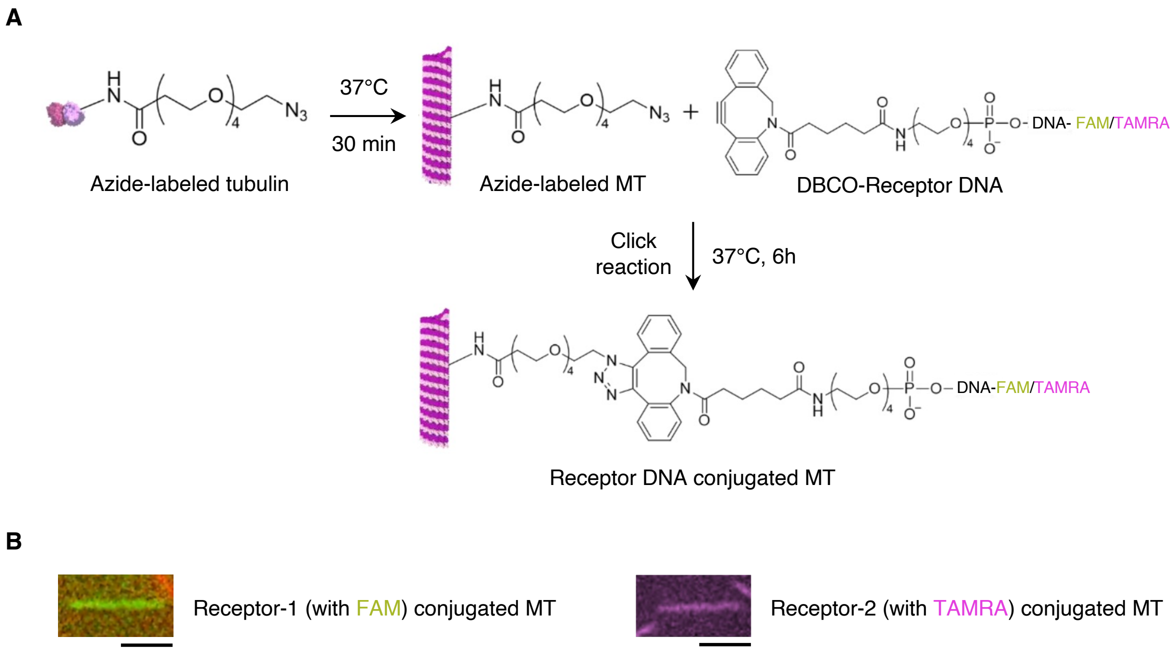

Fig. S5. Design and preparation of DNA-conjugated MTs. (**A**) Schematic diagram of the preparation of DNA-conjugated MTs through conjugation of MT with DNA by copper-free click reaction followed by polymerization of azide-labeled tubulins into azide-labeled MTs at 37 °C. (**B**) Fluorescence microscopy images of magenta and green receptor DNA-conjugated MTs. Scale bar: 5 µm.

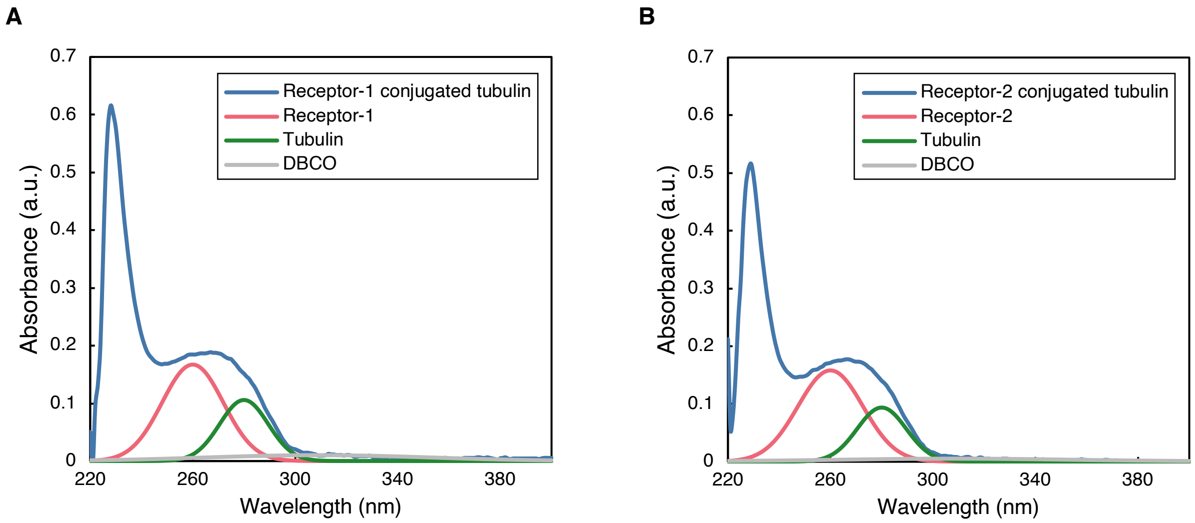

Fig. S6. UV-Vis absorption spectrum of DNA and tubulin. (**A**, **B**) Receptor-1 (A) and receptor-2 (B) conjugated MTs and evaluation of absorbance peaks of receptor strands, tubulin, and DBCO by Gaussian distribution function (a.u. = arbitrary unit). The concentrations of receptor strands were 500 µM in both cases. The estimated labeling ratios are given in table S2.

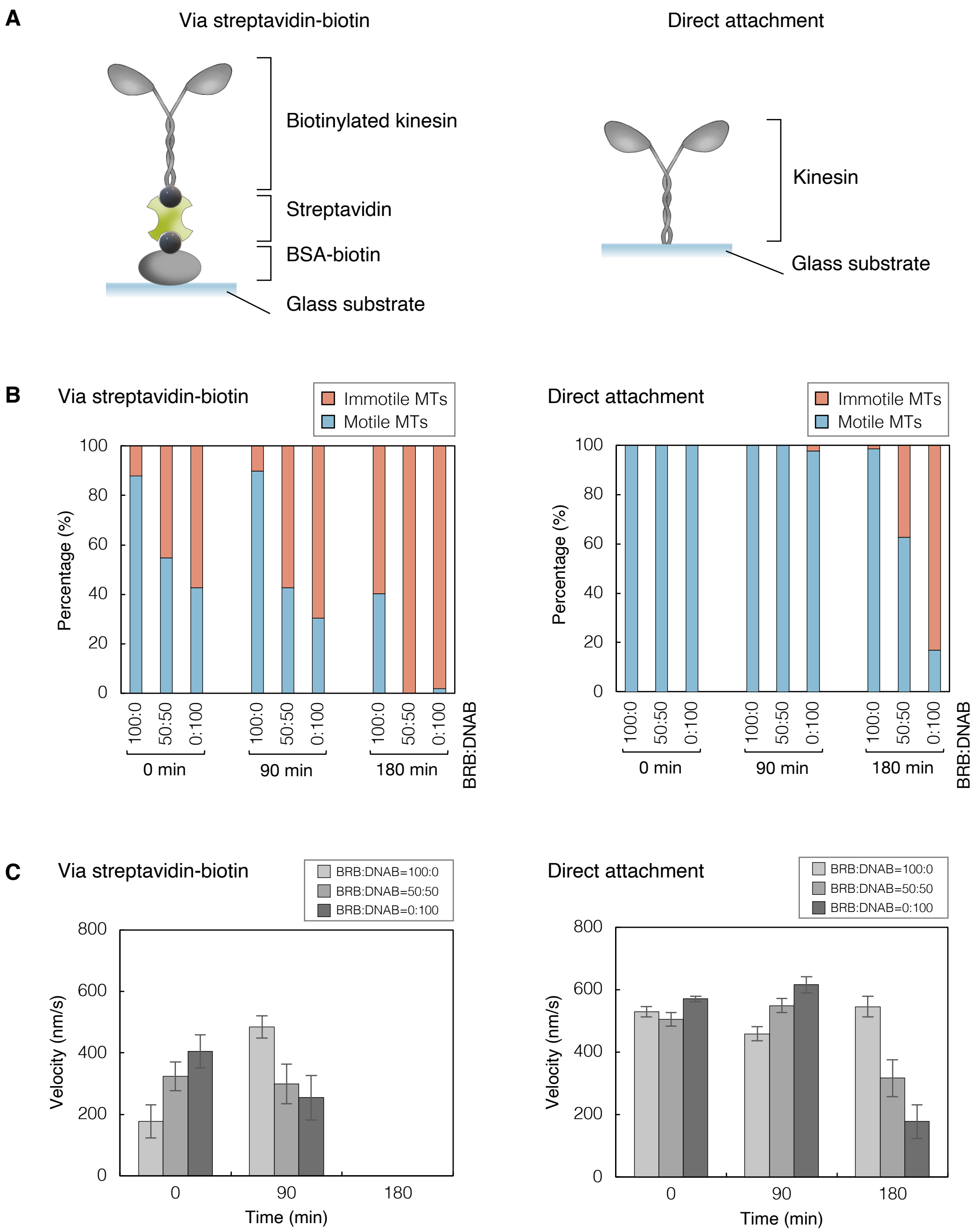

Fig. S7. Effect of surface modification on the resistance against salinity. (**A**) Two different glass surfaces with attaching kinesins. (left) Kinesins are attached using streptavidin-biotin interaction. (right) Kinesins are directly attached to the glass substrate. See Supplementary Method for a detailed experimental procedure. (**B**) Percentages of motile and immotile MTs after incubating with BRB and DNA-reaction buffer (DNAB). (**C**) Mean velocities of motile MTs (n = 30). The detailed experimental method is described in Supplementary Text 1.

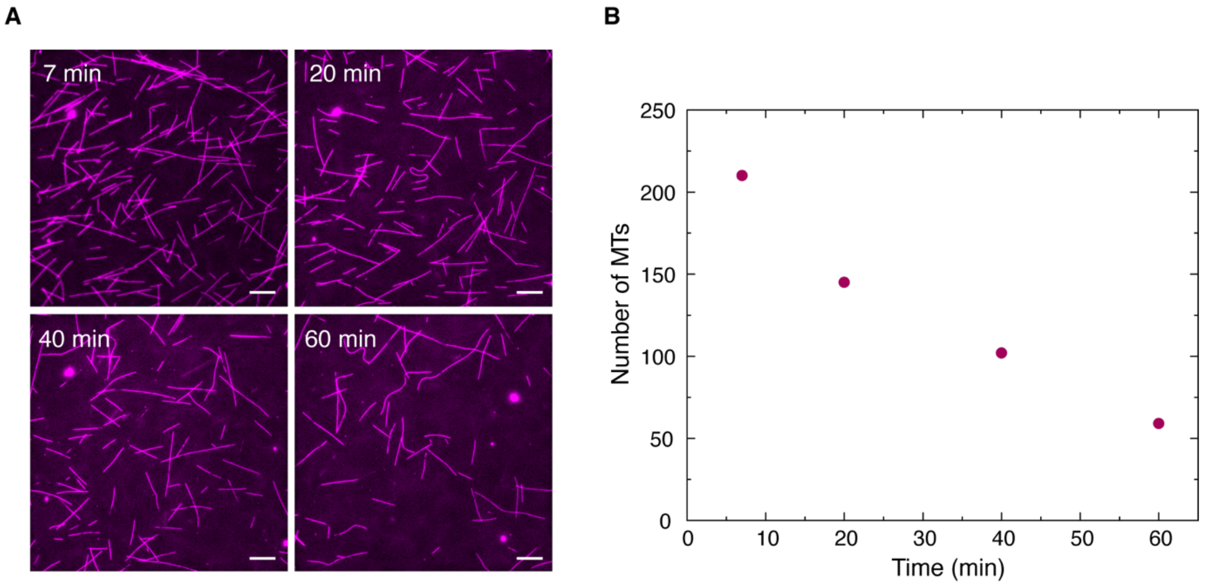

Fig. S8. Gliding assay of receptor-2 conjugated MTs under high-salinity and high-temperature condition. (**A**) Microscopy observation of the gliding assay of receptor-2 conjugated MTs in DNA reaction buffer at 37 °C. Scale bar: 25 μm. (**B**) The changes of the number of MTs over time. The number of MTs on a kinesin-coated surface decreases drastically in high salinity buffer (DNA reaction buffer) and at high temperature (37 °C). This is probably because of the detachment of MTs from the surface due to the hindrance of interaction between MTs and kinesins. Receptor-1 conjugated MTs also detaches similarly in this condition.

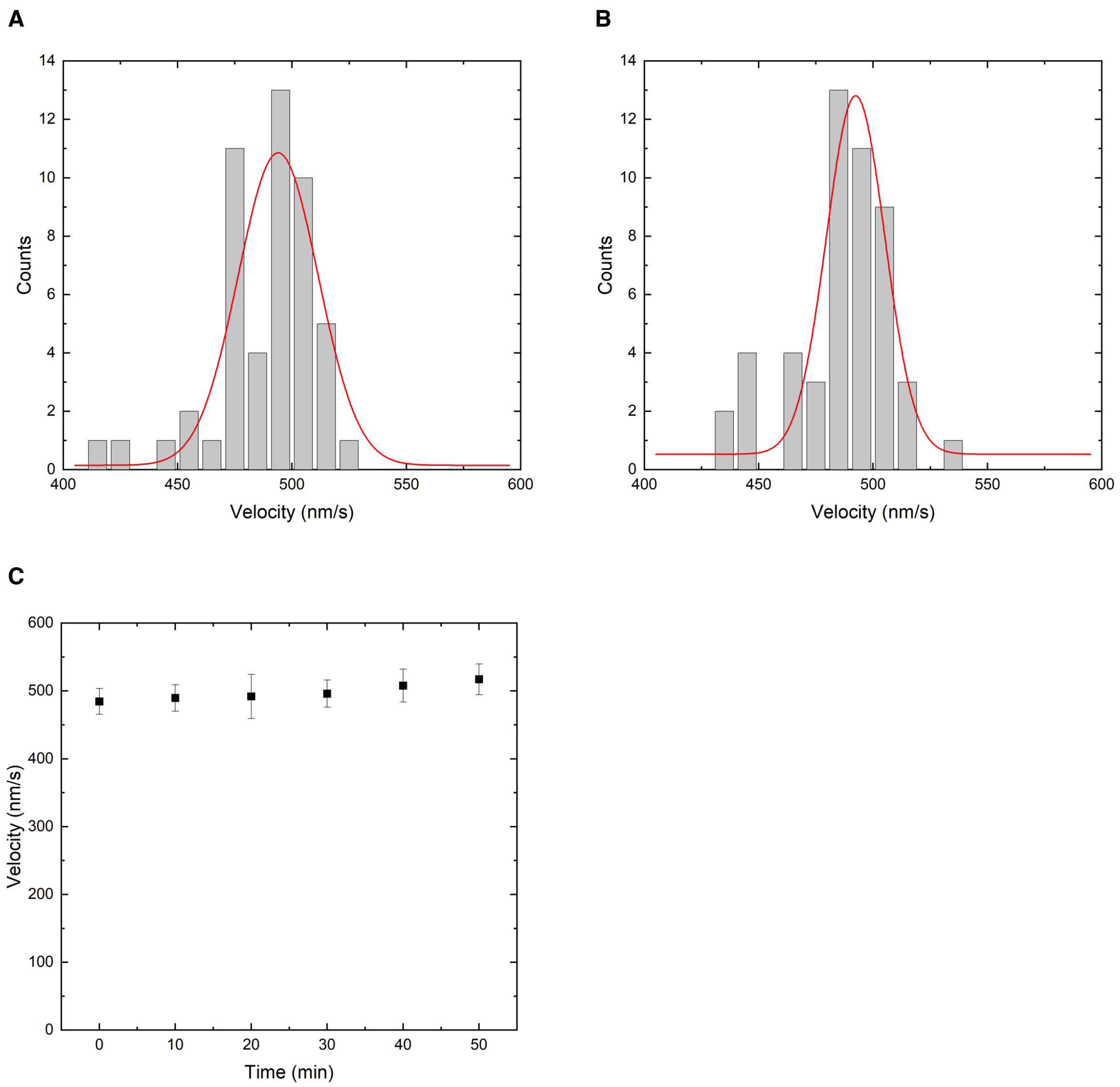

Fig. S9. Mean velocities of DNA-conjugated MTs. (**A**, **B**) Distribution of the velocities of Receptor-1 (A) and Receptor-2 (B) conjugated MTs. The number of MTs considered for analysis was 50 in both cases. The distributions of the velocities were fitted to the Gaussian equation ($y = y_0 + \frac{A}{w\sqrt{\pi/2}} e^{-2\frac{(x-x_c)^2}{w^2}}$) for normal distribution. The fitted curves are represented by the solid lines (R^2 = 0.78 and 0.88 for receptor-1 and receptor-2 conjugated MTs, respectively). The mean velocities obtained from the fitting were 490 ± 20 μm s^{-1} for both receptor-1 and receptor-2 conjugated MTs. The arithmetic means of the velocities of receptor-1 and receptor-2 conjugated MTs were both 490 ± 20 μm s^{-1}. (C) Mean velocities of discrete MTs over time (n = 30).

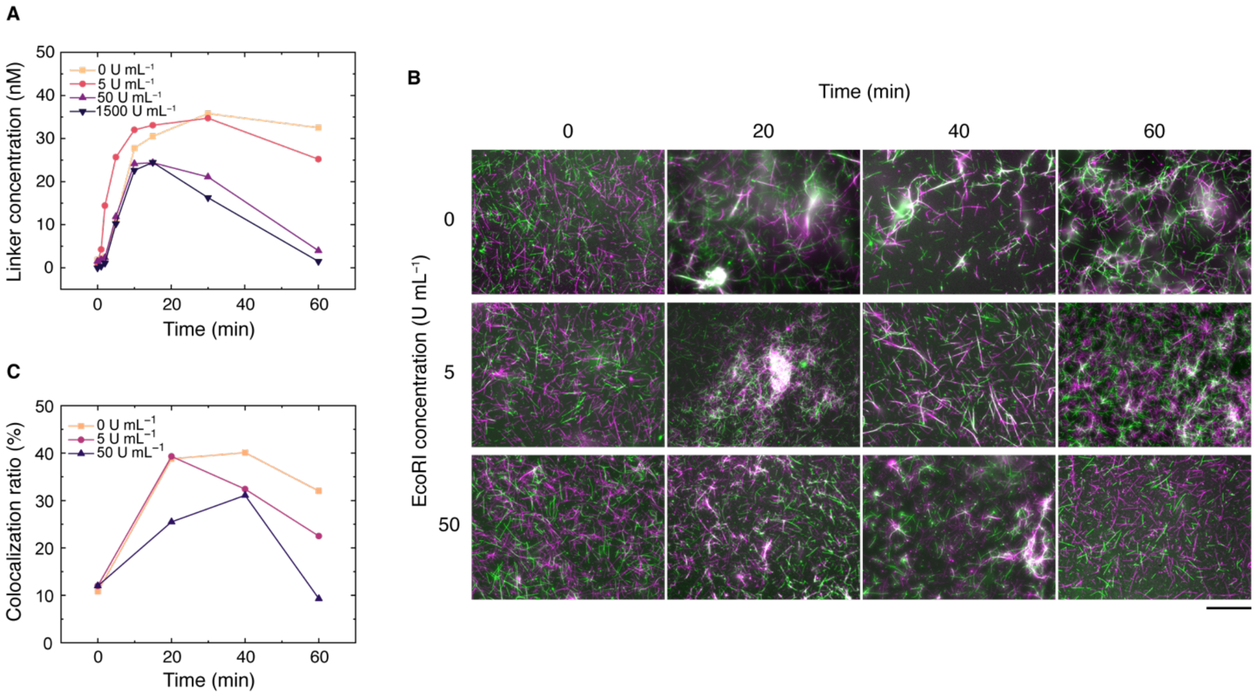

Fig. S10. Programmable assembly and disassembly of MTs by varying enzyme concentration. Because EcoRI only affects the third step of the DNA chemical reaction system, the dissociation time of MTs can be tuned. (**A**) Graph showing the time development of the linker concentrations quantified from gel electrophoresis. Results of experiments using final concentration of 0, 5, and 50 U mL^{-1} EcoRI is superimposed with the data of Fig. 2 (1500 U mL^{-1}). To obtain the data, the same experimental method of Fig. 2, and fig. S2 was used. (**B**) Snapshots of fluorescence microscopy, where we mixed DNA/enzymes solutions (incubated for 0, 20, 40, or 60 minutes) with green and magenta MTs on a kinesin-coated glass surface. Instead of a continuous time lapse imaging of the same sample, samples of each time point were observed separately by preparing flesh MT system each time. The DNA/enzymes samples were prepared similar to (A) by varying the incubation time and EcoRI concentration. Instead of using fluorescent labelled linker used in the gel electrophoresis for (A), we used unlabeled linker for this microscopy observation. MTs were prepared as described in the material and method of the main text. To obtain the images in this figure, IX83 Olympus, Japan was employed using 60x oil emulsion lens. Scale bar: 30 µm. (**C**) Time development of colocalization ratios quantified from the data of (B). As the concentration of EcoRI increased, the decrease of colocalization ratio became earlier.

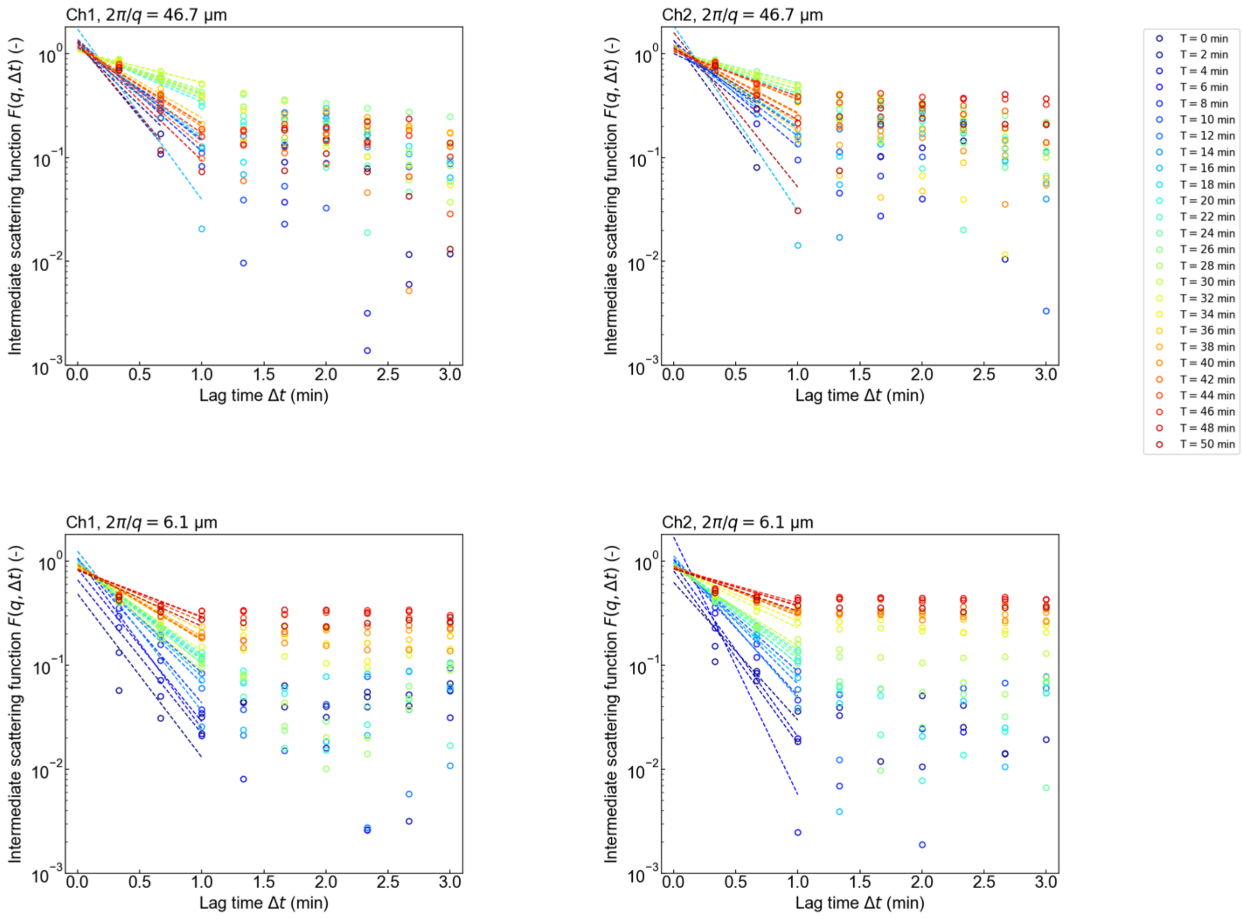

Fig. S11. Intermediate scattering function $F(q, \Delta t)$ plotted against lag time Δt at various elapsed time T obtained from DDM analysis. Ch1 and 2 represent green and magenta channels, respectively. Plots are fitted with exponential functions to determine the initial relaxation time τ. See Methods for details.

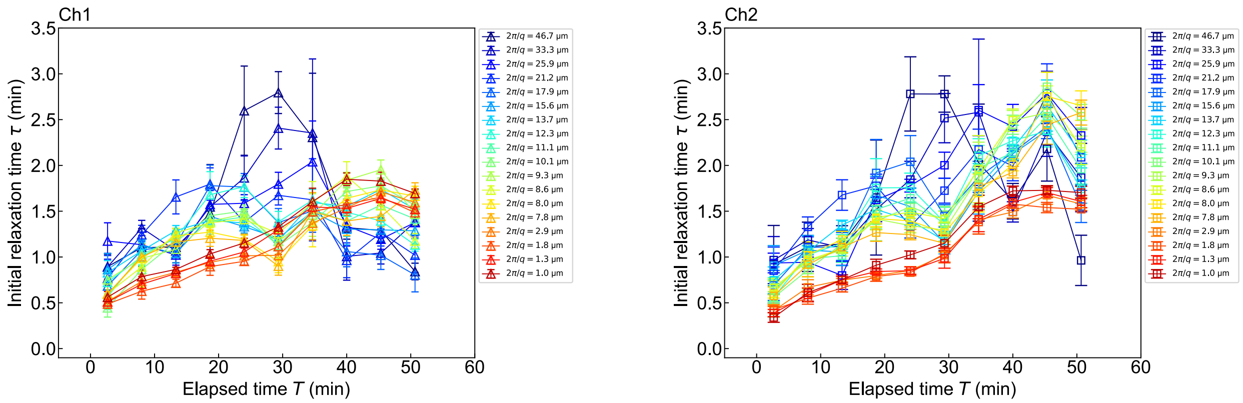

Fig. S12. Initial relaxation time τ plotted against elapsed time T under various wavelengths obtained from DDM analysis. Ch1 and 2 represent green and magenta channels, respectively. See Methods for details.

Table S1. Sequences of DNAs.

DNA	Sequences (5'-3')	5' end	3' end
Template-red	CTAGAACATACGCATTTGATTCGCCGGGAATTC TATAGTACTATTCAAGCGACGGCT	-	-
Template-gray	TCACTTCTGCATCGAGCTGAGGAGCCGTCGCTT GAATAGTGTTA	-	Phos
Template-orange	AGGATATGCTCTCGTGCTGAGGCCCGGCGAAT CAAATGCGTATGGTTA	-	Phos
Signal	TCAGCTCGATGCAGAAGTGA	-	-
Linker	TGGTATGTCAAGCCGCGAGACTGGCTGGTGAA GGATATGCTCTCGT	-	FAM
Converter-dark	ACGAGAGCATATCCTTCACCAGCCAGTCTCGC GGCTTGACATACCATCACTTCTGCATCGA	-	Phos
Updater	GGAGCCGTCGCTTGAATAGTACTATAGAATTC CCGATGACGCT	-	Phos
Transducer-blue	AGCGTCATCGGTCTTTATATAGTACTATTCAAG CGACGGCTTCACTTCTGC	-	-
Dissociator	TCAGCACGAGAGCATATCCT	-	-
Receptor-1	TTTTTTTTTTTTGCGGCTTGACATACCA	DBCO	FAM
Receptor-2	TTTTTTTTTTTTTCACCAGCCAGTCTGTTA	DBCO	TAMRA
Beacon-1	TCACTTCTGCATCGAGCTGATTTCGATGCAGA	-	TAMRA
Beacon-2	AGGATATGCTCTCGTGCTGATTACGAGAGCAT	-	TAMRA

All the DNAs were purified using HPLC.
Phos: 3'-Phosphorylated group. To inhibit extension by polymerase, phosphorylations were made at 3'end of receptors, template-gray, template-orange, linker, connector-dark, updater, and transducer-blue.

Table S2. Activities of enzymes in different buffer compositions and temperatures*.

Buffer Name	Unit	NEBuffer 2	NEBuffer 3.1	NEBuffer 4	BRB80 Na	BRB80 K	BRB80 Na/Mg	BRB80 K/Mg
Tris HCl	mM	10	10					
Tris acetate				20				
PIPES	mM				80	80	80	80
NaCl	mM	50	100					
CH_3COOK	mM			50				
NaOH	mM				165		165	
KOH	mM					165		165
$MgCl_2$	mM	10	10		1	1	10	10
$(CH_3COO)_2Mg$	mM			10				
EGTA	mM				1	1	1	1
DTT	mM	1		1				
BSA	µg/mL		100					
pH at 25℃		7.9	7.9	7.9	6.9	6.9	6.9	6.9
**Enzyme**	**T (°C)**	**NEBuffer 2**	**NEBuffer 3.1**	**NEBuffer 4**	**BRB80 Na**	**BRB80 K**	**BRB80 Na/Mg**	**BRB80 K/Mg**
Bst DNA Polymerase LargeFragment	25	50	64	88	90	84	106	114
	35	58	59	92	102	90	112	121
	45	61	63	101	103	100	114	124
Klenow Fragment (3'→5' exo-)	25	77	101	63	95	85	92	60
	35	68	102	58	96	88	97	76
	45	74	95	57	98	87	99	66
Nt.BstNBI	25	16	-18	37	-5	-1	-19	24
	35	34	0	40	-7	-2	-19	13
	45	50	6	43	-1	-3	-11	21
Nb.BbvCI	25	99	86	82	-4	22	23	54
	35	96	92	84	11	77	49	82
	45	100	93	84	39	87	58	86

*As buffer composition, we employed standard Tris buffers (NEBuffer2, NEBuffer3.1, NEBuffer4) from New England Biolabs, USA. and homemade BRB buffers (PIPES dissolved by NaOH or KOH in addition to 1 or 10 mM of $MgCl_2$). Enzymes used in reference 47 were Bst DNA polymerase and Nt.BstNBI, which are suitable for Tris buffer and higher temperature (45 ℃). Because we were afraid that these enzymes were not suitable for kinesin/MT system that work in BRB buffer and relatively low temperature, we switched to Klenow Fragment and Nb.NbvCI in this work. The buffer composition is summarized in the top half of the table to illustrate the difference among the buffers. In the bottom half of the table, the activity of each enzyme is shown in % unit (background color is blue for 0 and red for 100). The values were calculated from the images of gel electrophoresis experiments dedicated for the quantification. Note that some of the activity became below 0 or above 100 due to the variance to measure the band intensity from the

images. Each sample that contains 100 nM DNA and enzyme was incubated under the specified temperature for 100 min and then subjected to 12% acrylamide gel. After applying 200V to the gel for 30 minutes, the gel was stained by SYBR GOLD purchased from Invitrogen, USA. The DNA sequences used to quantify the Bst DNA polymerase, was CATTCTGACGAG (primer) and CTCGTCAGAATGCTCGTCAGAAT (template). For Klenow Fragment, we used CGAGTCTGTT (primer) and AACAGACTCGAACAGACTCG (template). Nt.BstNBI was quantified using DNA duplex formed from AACAGACTCGAACAGACTCG and CGAGTCTGTTCGAGTCTGTT, whereas Nb.BbvCI used CACCCTCAGCAACTCC and GGAGTTGCTGAGGGTG. We decided our buffer composition for further experiment from the results.

Table S3. Labeling ratios of DNA to tubulin dimers.

DNA	Infeed conc. of receptor (μM)	Final conc. of tubulin dimers (μM)	Final conc. of receptor in tubulin dimers (μM)	Labeling ratio of receptor to tubulin dimers (%)
Receptor-1	500	9	6	68
Receptor-2	500	8	6	78

Movie S1.

Autonomous assembly and disassembly of gliding microtubules regulated by a molecular controller. Scale bar: 25 μm.

REFERENCES AND NOTES

1. G. H. Wadhams, J. P. Armitage, Making sense of it all: Bacterial chemotaxis. *Nat. Rev. Mol. Cell Biol.* **5**, 1024–1037 (2004).

2. P. M. Lledo, G. Gheusi, J. D. Vincent, Information processing in the mammalian olfactory system. *Physiol. Rev.* **85**, 281–317 (2005).

3. A. Pavlovič, How the sensory system of carnivorous plants has evolved. *Plant Commun.* **3**, 100462 (2022).

4. G. Z. Yang, J. Bellingham, P. E. Dupont, P. Fischer, L. Floridi, R. Full, N. Jacobstein, V. Kumar, M. McNutt, R. Merrifield, B. J. Nelson, B. Scassellati, M. Taddeo, R. Taylor, M. Veloso, Z. L. Wang, R. Wood, The grand challenges of science robotics. *Sci. Robot.* **3**, eaar7650 (2018).

5. M. Brambilla, E. Ferrante, M. Birattari, M. Dorigo, Swarm robotics: A review from the swarm engineering perspective. *Swarm Intell.* **7**, 1–41 (2013).

6. S. Kim, C. Laschi, B. Trimmer, Soft robotics: A bioinspired evolution in robotics. *Trends Biotechnol.* **31**, 287–294 (2013).

7. S. Murata, A. Konagaya, S. Kobayashi, H. Saito, M. Hagiya, Molecular robotics: A new paradigm for artifacts. *New Gener. Comput.* **31**, 27–45 (2013).

8. M. Hagiya, A. Konagaya, S. Kobayashi, H. Saito, S. Murata, Molecular robots with sensors and intelligence. *Acc. Chem. Res.* **47**, 1681–1690 (2014).

9. S. Murata, *Molecular Robotics: An Introduction* (Springer Singapore, 2022).

10. D. Y. Zhang, G. Seelig, Dynamic DNA nanotechnology using strand-displacement reactions. *Nat. Chem.* **3**, 103–113 (2011).

11. N. C. Seeman, H. F. Sleiman, DNA nanotechnology. *Nat. Rev. Mater.* **3**, 17068 (2018).

12. X. Pan, T. Kortemme, Recent advances in de novo protein design: Principles, methods, and applications. *J. Biol. Chem.* **296**, 100558 (2021).

13. P. W. K. Rothemund, Folding DNA to create nanoscale shapes and patterns. *Nature* **440**, 297–302 (2006).

14. S. M. Douglas, H. Dietz, T. Liedl, B. Högberg, F. Graf, W. M. Shih, Self-assembly of DNA into nanoscale three-dimensional shapes. *Nature* **459**, 414–418 (2009).

15. S. M. Douglas, A. H. Marblestone, S. Teerapittayanon, A. Vazquez, G. M. Church, W. M. Shih, Rapid prototyping of 3D DNA-origami shapes with caDNAno. *Nucleic Acids Res.* **37**, 5001–5006 (2009).

16. Y. Suzuki, I. Kawamata, K. Watanabe, E. Mano, Lipid bilayer-assisted dynamic self-assembly of hexagonal DNA origami blocks into monolayer crystalline structures with designed geometries. *iScience* **25**, 104292 (2022).

17. Y. Xing, E. Cheng, Y. Yang, P. Chen, T. Zhang, Y. Sun, Z. Yang, D. Liu, Y. Xing, Z. Yang, D. Liu, E. Cheng, Y. Yang, Y. Sun, P. Chen, T. Zhang, Self-assembled DNA hydrogels with designable thermal and enzymatic responsiveness. *Adv. Mater.* **23**, 1117–1121 (2011).

18. R. Merindol, G. Delechiave, L. Heinen, L. H. Catalani, A. Walther, Modular design of programmable mechanofluorescent DNA hydrogels. *Nat. Commun.* **10**, 528 (2019).

19. N. Srinivas, J. Parkin, G. Seelig, E. Winfree, D. Soloveichik, Enzyme-free nucleic acid dynamical systems. *Science* **358**, eaal2052 (2017).

20. T. Rodjanapanyakul, F. Takabatake, K. Abe, I. Kawamata, S. M. Nomura, S. Murata, Diffusion modulation of DNA by toehold exchange. *Phys. Rev. E.* **97**, 052617 (2018).

21. K. Abe, S. Murata, I. Kawamata, Cascaded pattern formation in hydrogel medium using the polymerisation approach. *Soft Matter* **17**, 6160–6167 (2021).

22. T. Fu, Y. Lyu, H. Liu, R. Peng, X. Zhang, M. Ye, W. Tan, DNA-based dynamic reaction networks. *Trends Biochem. Sci.* **43**, 547–560 (2018).

23. H. Gu, J. Chao, S. J. Xiao, N. C. Seeman, A proximity-based programmable DNA nanoscale assembly line. *Nature* **465**, 202–205 (2010).

24. K. Lund, A. J. Manzo, N. Dabby, N. Michelotti, A. Johnson-Buck, J. Nangreave, S. Taylor, R. Pei, M. N. Stojanovic, N. G. Walter, E. Winfree, H. Yan, Molecular robots guided by prescriptive landscapes. *Nature* **465**, 206–210 (2010).

25. Y. Suzuki, I. Kawamata, K. Mizuno, S. Murata, Large deformation of a DNA-origami nanoarm induced by the cumulative actuation of tension-adjustable modules. *Angewandte. Chemie Int. Ed.* **59**, 6230–6234 (2020).

26. A. Padirac, T. Fujii, Y. Rondelez, Bottom-up construction of in vitro switchable memories. *Proc. Natl. Acad. Sci. U.S.A.* **109**, E3212–E3220 (2012).

27. T. Fujii, Y. Rondelez, Predator–Prey molecular ecosystems. *ACS Nano* **7**, 27–34 (2013).

28. A. Cangialosi, C. Yoon, J. Liu, Q. Huang, J. Guo, T. D. Nguyen, D. H. Gracias, R. Schulman, DNA sequence–directed shape change of photopatterned hydrogels via high-degree swelling. *Science* **357**, 1126–1130 (2017).

29. H. Dehne, A. Reitenbach, A. R. Bausch, Reversible and spatiotemporal control of colloidal structure formation. *Nat. Commun.* **12**, 6811 (2021).

30. H. Fang, M. F. Hagan, W. Benjamin Rogers, Two-step crystallization and solid–solid transitions in binary colloidal mixtures. *Proc. Natl. Acad. Sci. U.S.A.* **117**, 27927–27933 (2020).

31. G. Saper, H. Hess, Synthetic systems powered by biological molecular motors. *Chem. Rev.* **120**, 288–309 (2020).

32. J. Howard, A. J. Hunt, S. Baek, Chapter 10 Assay of microtubule movement driven by single kinesin molecules. *Methods Cell Biol.* **39**, 137–147 (1993).

33. T. Nitta, Y. Wang, Z. Du, K. Morishima, Y. Hiratsuka, A printable active network actuator built from an engineered biomolecular motor. *Nat. Mater.* **20**, 1149–1155 (2021).

34. S. Chaudhuri, T. Korten, S. Korten, G. Milani, T. Lana, G. Te Kronnie, S. Diez, Label-free detection of microvesicles and proteins by the bundling of gliding microtubules. *Nano Lett.* **18**, 117–123 (2018).

35. D. V. Nicolau, M. Lard, T. Korten, F. C. M. J. M. Van Delft, M. Persson, E. Bengtsson, A. Månsson, S. Diez, H. Linke, D. V. Nicolau, Parallel computation with molecular-motor-propelled agents in nanofabricated networks. *Proc. Natl. Acad. Sci. U.S.A.* **113**, 2591–2596 (2016).

36. A. M. R. Kabir, D. Inoue, A. Kakugo, Molecular swarm robots: Recent progress and future challenges. *Sci. Technol. Adv. Mater.* **21**, 323–332 (2020).

37. K. Matsuda, A. M. R. Kabir, N. Akamatsu, A. Saito, S. Ishikawa, T. Matsuyama, O. Ditzer, M. S. Islam, Y. Ohya, K. Sada, A. Konagaya, A. Kuzuya, A. Kakugo, Artificial smooth muscle model composed of hierarchically ordered microtubule asters mediated by DNA origami nanostructures. *Nano Lett.* **19**, 3933–3938 (2019).

38. Y. Vyborna, J. C. Galas, A. Estevez-Torres, DNA-controlled spatiotemporal patterning of a cytoskeletal active gel. *J. Am. Chem. Soc.* **143**, 20022–20026 (2021).

39. A. M. Tayar, M. F. Hagan, Z. Dogic, Active liquid crystals powered by force-sensing DNA-motor clusters. *Proc. Natl. Acad. Sci. U.S.A.* **118**, e2102873118 (2021).

40. Y. Sato, Y. Hiratsuka, I. Kawamata, S. Murata, S. M. Nomura, Micrometer-sized molecular robot changes its shape in response to signal molecules. *Sci. Robot.* **2**, eaal3735 (2017).

41. A. J. M. Wollman, C. Sanchez-Cano, H. M. J. Carstairs, R. A. Cross, A. J. Turberfield, Transport and self-organization across different length scales powered by motor proteins and programmed by DNA. *Nat. Nanotechnol.* **9**, 44–47 (2014).

42. A. Senoussi, J. C. Galas, A. Estevez-Torres, Programmed mechano-chemical coupling in reaction-diffusion active matter. *Sci. Adv.* **7**, eabi9865 (2021).

43. J. J. Keya, A. M. R. Kabir, D. Inoue, K. Sada, H. Hess, A. Kuzuya, A. Kakugo, Control of swarming of molecular robots. *Sci. Rep.* **8**, 11756 (2018).

44. J. J. Keya, R. Suzuki, A. M. R. Kabir, D. Inoue, H. Asanuma, K. Sada, H. Hess, A. Kuzuya, A. Kakugo, DNA-assisted swarm control in a biomolecular motor system. *Nat. Commun.* **9**, 453 (2018).

45. S. Ishii, M. Akter, K. Murayama, A. M. R. Kabir, H. Asanuma, K. Sada, A. Kakugo, Kinesin motors driven microtubule swarming triggered by UV light. *Polym. J.* **54**, 1501–1507 (2022).

46. M. Akter, J. J. Keya, K. Kayano, A. M. R. Kabir, D. Inoue, H. Hess, K. Sada, A. Kuzuya, H. Asanuma, A. Kakugo, Cooperative cargo transportation by a swarm of molecular machines. *Sci. Robot.* **7**, eabm0677 (2022).

47. I. Kawamata, S. M. Nomura, S. Murata, Autonomous and programmable strand generator implemented as DNA and enzymatic chemical reaction cascade. *New Gener. Comput.* **40**, 723–736 (2022).

48. D. Y. Zhang, E. Winfree, Control of DNA strand displacement kinetics using toehold exchange. *J. Am. Chem. Soc.* **131**, 17303–17314 (2009).

49. J. N. Zadeh, C. D. Steenberg, J. S. Bois, B. R. Wolfe, M. B. Pierce, A. R. Khan, R. M. Dirks, N. A. Pierce, NUPACK: Analysis and design of nucleic acid systems. *J. Comput. Chem.* **32**, 170–173 (2011).

50. B. Mickey, J. Howard, Rigidity of microtubules is increased by stabilizing agents. *J. Cell Biol.* **130**, 909–917 (1995).

51. F. Giavazzi, D. Brogioli, V. Trappe, T. Bellini, R. Cerbino, Scattering information obtained by optical microscopy: Differential dynamic microscopy and beyond. *Phys. Rev. E.* **80**, 031403 (2009).

52. L. G. Wilson, V. A. Martinez, J. Schwarz-Linek, J. Tailleur, G. Bryant, P. N. Pusey, W. C. K. Poon, Differential dynamic microscopy of bacterial motility. *Phys. Rev. Lett.* **106**, 018101 (2011).

53. J. Kim, E. Winfree, Synthetic in vitro transcriptional oscillators. *Mol. Syst. Biol.* **7**, 465 (2011).

54. S. B. Rivera, S. J. Koch, J. M. Bauer, J. M. Edwards, G. D. Bachand, Temperature dependent properties of a kinesin-3 motor protein from Thermomyces lanuginosus. *Fungal Genet. Biol.* **44**, 1170–1179 (2007).

55. S. Okumura, G. Gines, A. Baccouche, R. Deteix, T. Fujii, Y. Rondelez, A. J. Genot, Nonlinear decision-making with enzymatic neural networks. *Nature* **610**, 496–501 (2022).

56. M. Castoldi, A. V. Popov, Purification of brain tubulin through two cycles of polymerization-depolymerization in a high-molarity buffer. *Protein Expr. Purif.* **32**, 83–88 (2003).

57. R. B. Case, D. W. Pierce, N. Hom-Booher, C. L. Hart, R. D. Vale, The directional preference of kinesin motors is specified by an element outside of the motor catalytic domain. *Cell* **90**, 959–966 (1997).

58. J. Peloquin, Y. Komarova, G. Borisy, Conjugation of fluorophores to tubulin. *Nat. Methods* **2**, 299–303 (2005).

59. S. M. Früh, D. Steuerwald, U. Simon, V. Vogel, Covalent cargo loading to molecular shuttles via copper-free "click chemistry." *Biomacromolecules* **13**, 3908–3911 (2012).

60. S. V. Costes, D. Daelemans, E. H. Cho, Z. Dobbin, G. Pavlakis, S. Lockett, Automatic and quantitative measurement of protein-protein colocalization in live cells. *Biophys. J.* **86**, 3993–4003 (2004).

61. P. Edera, D. Bergamini, V. Trappe, F. Giavazzi, R. Cerbino, Differential dynamic microscopy microrheology of soft materials: A tracking-free determination of the frequency-dependent loss and storage moduli. *Phys. Rev. Mater.* **1**, 073804 (2017).

62. R. McGorty, PyDDM v0.2.0 (2022); https://doi.org/10.5281/zenodo.6626403.

63. H. N. Verwei, G. Lee, G. Leech, I. I. Petitjean, G. H. Koenderink, R. M. Robertson-Anderson, R. J. McGorty, Quantifying cytoskeleton dynamics using differential dynamic microscopy. *J. Vis. Exp.* **184**, e63931 (2022).

64. K. Fujimoto, M. Kitamura, M. Yokokawa, I. Kanno, H. Kotera, R. Yokokawa, Colocalization of quantum dots by reactive molecules carried by motor proteins on polarized microtubule arrays. *ACS Nano* **7**, 447–455 (2013).
